# Polyphenol profile by UHPLC-MS/MS, anti-glycation, antioxidant and cytotoxic activities of several samples of propolis from the northeastern semi-arid region of Brazil

**DOI:** 10.1080/13880209.2017.1340962

**Published:** 2017-06-20

**Authors:** Jadriane de Almeida Xavier, Iara Barros Valentim, Fabiana O. S. Camatari, Alberto M. M. de Almeida, Henrique Fonseca Goulart, Jamylle Nunes de Souza Ferro, Emiliano de Oliveira Barreto, Bruno Coelho Cavalcanti, Carla B. G. Bottoli, Marília Oliveira Fonseca Goulart

**Affiliations:** a Instituto de Química e Biotecnologia, Universidade Federal de Alagoas (UFAL), Maceió, AL, Brazil;; b Instituto Federal de Educação, Ciência e Tecnologia de Alagoas (IFAL), Rua Mizael Domingues, Maceió, AL, Brazil;; c Empresa Baiana de Desenvolvimento Agrícola S.A (EBDA), Salvador, BA, Brazil;; d Laboratório de Pesquisas em Recursos Naturais, Centro de Ciências Agrárias (CECA), UFAL, Rio Largo, AL, Brazil;; e Laboratório de Biologia Celular, UFAL, Maceio, AL, Brazil;; f Departamento de Fisiologia e Farmacologia, Laboratório Nacional de Oncologia Experimental, Universidade Federal do Ceará, Fortaleza, CE, Brazil;; g Instituto de Química, Universidade de Campinas, Campinas, SP, Brazil

**Keywords:** biologically active phenols, chemical profile, content of organic volatiles

## Abstract

**Context:** Propolis has promising biological activities. Propolis samples from the Northeast of Bahia, Brazil – sample A from Ribeira do Pombal and B, from Tucano – were investigated, with new information regarding their biological activities.

**Objective:** This paper describes the chemical profile, antioxidant, anti-glycation and cytotoxic activities of these propolis samples.

**Material and methods:** Ethanol extracts of these propolis samples (EEP) and their fractions were analyzed to determine total phenolic content (TPC); antioxidant capacity through DPPH^•^, FRAP and lipid peroxidation; anti-glycation activity, by an *in vitro* glucose (10 mg/mL) bovine serum albumine (1 mg/mL) assay, during 7 d; cytotoxic activity on cancer (SF295, HCT-116, OVCAR-8, MDA-MB435, MX-1, MCF7, HL60, JURKAT, MOLT-4, K562, PC3, DU145) and normal cell lines (V79) at 0.04–25 μg/mL concentrations, for 72 h. The determination of primary phenols by ultra high-pressure liquid chromatography coupled to tandem mass spectrometry (UHPLC-MS/MS) and volatile organic compounds content by gas chromatography-mass spectrometry (GC-MS) were also performed.

**Results:** The EEP polar fractions exhibited up to 90% protection against lipid peroxidation. The IC_50_ value for anti-glycation activity of EEP was between 16.5 and 19.2 μg/mL, close to aminoguanidine (IC_50_ = 7.7 μg/mL). The use of UHPLC-MS/MS and GC-MS allowed the identification of 12 bioactive phenols in the EEP and 24 volatile compounds, all already reported.

**Conclusions:** The samples present good antioxidant/anti-glycation/cytotoxic activities and a plethora of biologically active compounds. These results suggest a potential role of propolis in targeting ageing and diseases associated with oxidative and carbonylic stress, aggregating value to them.

## Introduction

Propolis is a natural gummy and balsamic resin, obtained from resinous substances, collected by honey bees from flowers, buds and plant exudates. It has been attracting scientific attention due to its biological and pharmacological properties, which are related to its chemical composition (Silva-Carvalho et al. 2015). It varies according to the botanical origin of the resinous substances, season of the year and environmental conditions at the site of collection. For these reasons, there are many different types of propolis, with considerable chemical diversity (Huang et al. 2014). The standardization of propolis with respect to its chemical composition is difficult (Silva-Carvalho et al. 2015), but it is urgently required.

Food, nutraceuticals and other products rich in antioxidants can protect an organism against reactive oxygen species (ROS) and advanced glycation/lipoxidation end product (AGE/ALE) accumulation (Boisard et al. 2014). AGEs and ALEs are formed through specific condensation reactions between nucleophiles, like amino groups of free amino acids or their residues in peptides, aminophospholipids or proteins, and electrophiles, such as oxidized products from excess ROS, for instance, carbonyls of reducing sugars, oxidized lipids and/or others, generating well-defined sets of covalent adducts (Chinchansure et al. 2015; Barbosa et al. 2016). The adverse role of these AGE precursors is observed in a wide spectra of pathogenic conditions, including microvascular and macrovascular diseases such as nephropathy, retinopathy, peripheral neuropathy and arteriosclerosis in diabetes mellitus (Lo et al. 2006; Uribarri et al. 2015). The identification of anti-glycation agents to prevent the formation of these compounds holds great promise as they can be used for supplementary treatment of the complications of diabetes mellitus, such as microangiopathy or microneuropathy (Lo et al. 2006; Ramkissoon et al. 2012a; Uribarri et al. 2015). Much effort has been expended to search for dietary plants that can effectively inhibit AGE formation that also have antioxidant properties (Ramkissoon et al. 2012a, 2012b). Compounds offering both properties have been reported to show greater efficacy for treating diabetes mellitus versus compounds targeting an individual pathway (Duraisamy et al. 2003). As such, on-going screening of natural compounds that offer combined antioxidant and anti-glycation properties with relatively low toxicity are promising candidates for the development of functional additives aimed at reducing protein glycation for the treatment and management of oxidative stress-related diseases, diabetic complications and other AGE-associated diseases (Elosta et al. 2012; Ramkissoon et al. 2012a, 2012b, 2013; Sahebi and Divsalar 2016). In addition, a few studies report the ability of propolis to prevent fluorescent AGE formation (Oršolić et al. 2012; Boisard et al. 2014; Sahebi and Divsalar 2016).

In Brazil, due to its wide biodiversity that produces different biological properties and different chemical compositions of propolis, many uses have been reported (Pereira et al. 2002). Propolis has been used as a food supplement and as a source of bioactive compounds, including polyphenols, flavonoid aglycones, phenolic acids and their esters, as well as phenolic aldehydes and ketones (Toreti et al. 2013; Huang et al. 2014). Some studies indicate that propolis exhibits antitumor activity and can be used, for instance, in the treatment of skin cancer, lung cancer and tumors of the throat and brain (Slavov et al. 2014). The ethanolic extract of red propolis has been able to inhibit the growth of cancerous cells of human laryngeal epidermoid carcinoma cells (Hep-2) and human cervical adenocarcinoma (HeLa) (Frozza et al. 2013). Eleven chemical components isolated from the water extract of Chinese propolis were tested using human tumor cell lines of breast (MCF-7, MDA-MB-231), lung (A549) and HeLa. Chemical constituents from propolis: pinobanksin, caffeic acid benzyl ester, caffeic acid phenethyl ester, apigenin, pinocembrin, chrysin and galangin significantly inhibited the proliferation of four tumor cell lines (Xuan et al. 2016).

Brazilian propolis is classified into 13 different groups according to their geographical origin, chemical composition and vegetal source. Park et al. (2000) classified types according to their appearance, coloration, UV–VIS absorption spectra and chemical profile using thin-layer chromatography, high-performance chromatography and biological properties. However, the chemical profile analysis was performed only comparing differences in the chromatograms obtained, without identification of compounds, or chemical markers for each type of propolis. Thus, it is important to study and characterize samples produced in distinct regions according to their chemical markers (Toreti et al. 2013).

In the present study, two propolis samples from the Northeast of Bahia, referred as sample A from the Ribeira do Pombal region, and sample B, from Tucano, were investigated for their chemical profiles, antioxidant and anti-glycation capacities and cytotoxic activity against several cancer cell lines, in comparison with normal cell lines. To our knowledge, this is the first report on propolis from these regions. The anti-glycation properties of these propolis samples also, up to now have not been examined.

## Materials and methods

### Chemicals

Folin–Ciocalteau (FC) reagent, ethanol, methanol, 2,2′-diphenyl-1-picrylhydrazyl radical (DPPH^•^), 2,4,6-tripyridyl-s-triazine (TPTZ), 2,2-azobis(2-amidinopropane) dihydrochloride (AAPH), apigenin, caffeic acid, cinnamic acid, coumaric acid, chlorogenic acid, ferulic acid, gallic acid, sinapic acid, syringic acid, vanillic acid, kaempferol, catechin, epicatechin, epigallocatechin, formononetin, luteolin, quercetin, rutin, 3,4-dihydroxybenzoic acid, 3-(4,5-dimethylthiazol-2-yl)-2,5-diphenyltetrazolium bromide (MTT) and Trolox^®^ were purchased from Sigma Aldrich (Steinheim, Germany) and used as received. Sodium carbonate and DMSO (dimethylsulfoxide) were supplied by Vetec Química Fina Ltda (Rio de Janeiro, Brazil) and Trolox^®^ by Merck (Düsseldorf, Germany). 4,4-Difluoro-5-(4-phenyl-1,3-butadienyl)-4-bora-3a,4a-diaza-*S*-indacene-3-undecanoic acid (C11-BODIPY_581/591_) was obtained from Molecular Probes (Ontario, Canada). All reagents were of analytical grade and the stock solutions and buffers were prepared with Milli-Q purified water.

### Propolis samples

Samples of crude propolis produced by *Apis mellifera* bees were collected in May 2014 from Ribeira do Pombal (10°91′S; 38°41′W; 245.50 m, sample A) and Tucano (11°19′S; 38°70′W; 414.45 m, sample B) in the state of Bahia. The collection of samples was carried out by competent collectors from the beekeeper cooperative. The samples were stored in amber glass vials in a freezer until further use.

### Preparation of the ethanol extracts of propolis (EEP) and their fractions

**Figure 1.**
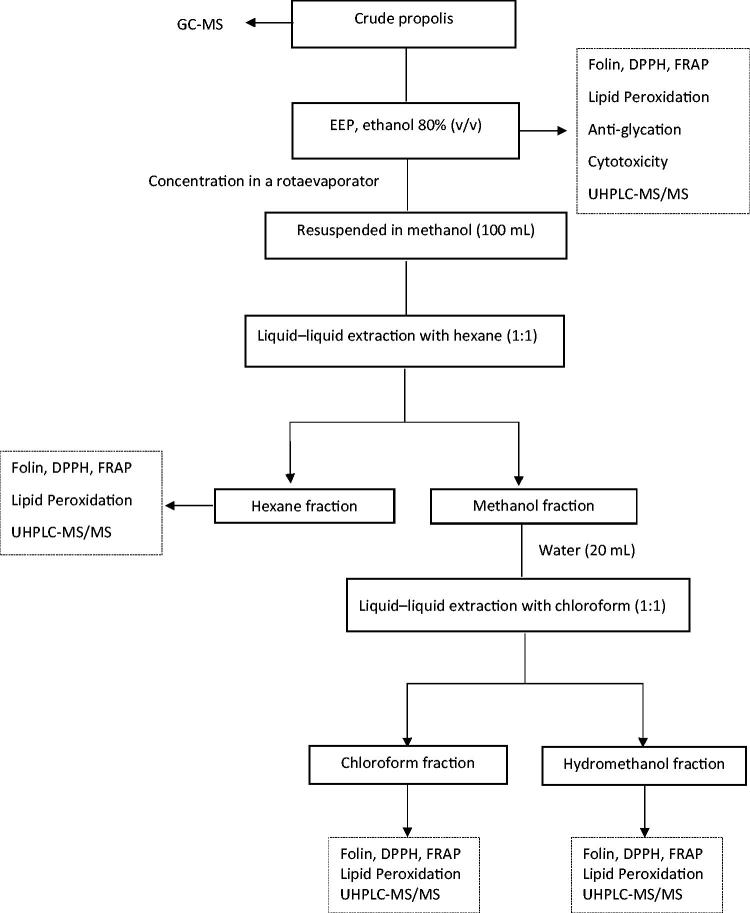
Simplified flow chart of the experiment performed with the two samples of propolis (EEPA and EEPB).

Propolis samples (8 g) were extracted with 80% (v/v) ethanol (100 mL) in a water bath at 70 °C for 30 min and, after filtration, concentrated in a rotaevaporator, resulting in dry extracts, designed as EEPA, from sample A, and EEPB, from sample B. These extracts were resuspended in methanol (100 mL). The methanol solution was further fractioned by liquid–liquid extraction with hexane and chloroform. For a better separation in CHCl_3_, water was added (20 mL). This treatment led to three fractions: a hexane fraction (Hex-fr), a chloroform fraction (Chlo-fr) and a hydromethanol fraction (HMet-fr). All extracts were concentrated in a rotaevaporator, adequately labeled and stored in amber glass vials under refrigeration. The experimental steps performed with the two propolis samples are displayed in Figure 1.

### Estimation of total polyphenolic content

The total phenolic contents (TPCs) of the extracts (EEPs and fractions) were determined using FC reagent, as described by Cicco et al. (2009) with the following modifications: aliquots (120 μL) of ethanolic solutions of propolis (25 μg/mL) were placed in test tubes, followed by the addition of 180 μL of water. About 300 μL of FC reagent were added to each tube. After 2 min, 2400 μL of a 5% (w/v) sodium carbonate solution were added. The mixture was shaken and heated at 40 °C in a water bath for 20 min. The tubes were then rapidly cooled and the developed color was read at 760 nm in a MultiSpec–1501 UV–Vis spectrophotometer (Shimadzu, Tokyo, Japan). The concentration of phenolic compounds was estimated using a calibration curve constructed with gallic acid (GA) in ethanol (0.7–7.0 mg/L) as a polyphenol reference (*n* = 3). The results are expressed as mg of GA equivalents/g dry extract. The same procedure was performed using 120 μL of water, as a blank.

### Radical scavenging ability toward DPPH^•^ (RSA-DPPH^•^)

The antioxidant capacities of the EEPs and fractions were measured in terms of their radical scavenging ability (RSA), using the DPPH^•^ method (Sánchez-Moreno et al. 1999). The residue obtained from 0.30 mL of the extract dissolved in ethanol was mixed with 2.70 mL of DPPH^•^ solution (40 μg/mL in methanol) to give a final sample concentration of approximately 25 μg/mL. The mixture was then homogenized and stored in the dark prior to analysis. The percentage of DPPH^•^ radical-scavenging activity (RSA% – DPPH^•^) of each sample was calculated as follows: RSA% = (1 − AC/AD) × 100, where AC is the absorbance of the solution when the sample was added at a particular level and AD is the absorbance of the original DPPH^•^ solution. The IC_50_ (half maximal inhibitory concentration) was calculated graphically, using a calibration curve, in the linear range by plotting the extract concentration *versus* the corresponding scavenging effect (I%, inhibition percentage), over 30 min. The value of I% was calculated using the equation: I% = [(A_0_ – A_1_)/A_0_] × 100, where A_0_ is the absorbance of the control and A_1_ the absorbance in the presence of the extract or fraction.

### FRAP assay

The assay was performed, according to the method described by Benzie and Strain (1996) with some modifications. In brief, the FRAP reagent was prepared by mixing 2.5 mL of a solution of TPTZ (10 mmol/L) in 40 mmol/L HCl, 2.5 mL of FeCl_3_ (20 mmol/L) and 25 mL of 0.30 mol/L acetate buffer (pH 3.6). Sample aliquots (90 μL) were mixed with 270 μL of distilled water and 2.7 mL of FRAP reagent and then incubated at 37 °C for 30 min, resulting in a final concentration of 25 μg/mL of EEPs or fractions. The absorbance of the reaction mixture was measured, at 595 nm, and a calibration curve was prepared with Trolox^^®^^ (0.04–7.50 mg/L). The results are expressed as trolox equivalent antioxidant capacities (TEAC), in mg of Trolox/g of dry extract.

### Lipid peroxidation measurements

Unilamellar vesicles of soy phosphatidylcholine (1 mmol/L) were prepared by extrusion (MacDonald et al. 1991) (100 nm pore diameter membrane, at 25 °C) in 10 mL of phosphate buffer (50 mmol/L), pH 7.4, with the additional incorporation of 0.1 μmol/L of the peroxyl-sensitive fluorescent probe C11-BODIPY_581/591_ (Drummen et al. 2002). The particle size was measured by Nanotrac-Zetatrac, NPA151-31A-0000-D30-10M model (Microtrac, York, PA), being around 100 nm.

Fluorescence measurements were carried out at 37 °C, using a RF-5301PC spectrofluorophotometer (Shimadzu, Tokyo, Japan). In a 1-mL quartz cuvette, adequate amounts of unilamellar vesicle suspension, phosphate buffer (pH 7.4) and the sample (EEPs or fractions) (final concentrations of 25 μg/mL) or positive controls (Trolox^®^, 100 μmol/L) were mixed. Ethanol and buffer were used as negative controls. The reaction was initiated with the addition of 100 μL of AAPH (100 mmol/L). The fluorescence decay (*λ*_exc_ = 580 nm, *λ*_em_ = 600 nm) was continuously monitored over 30 min.

### Anti-glycation activity

This assay was performed in triplicate according to the method described by Beaulieu et al. (2009) and Melo et al. (2015) with some modifications. Initially, the incubation medium consisting of BSA (1 mg/mL), glucose (10 mg/mL) and fructose (10 mg/mL) was prepared in sodium phosphate buffer (100 mmol/L pH 7.4). Seven different concentrations of ethanol solutions of the dry extracts of EEPA or EEPB (2.5–100 μg/mL) were analyzed to determine the IC_50_. To determine whether the EEP interfered with fluorescence, an extract blank containing glucose (100 mmol/L) and fructose (100 mmol/L) in 100 mmol/L phosphate buffer was prepared for each dilution. A negative control, containing glucose (10 mg/mL), fructose (10 mg/mL), BSA (1 mg/mL) and the vehicle in 100 mmol/L phosphate buffer and a positive control, containing aminoguanidine (0.78–50 μg/mL), were also prepared and assayed.

The samples were incubated in the dark at 37 °C with constant stirring for 7 d and the formation of AGE was quantified, using a RF-5301PC spectrofluorophotometer (Shimadzu, Tokyo, Japan) at *λ*_ex_ = 355 and *λ*_em_ = 440 nm. The analyses were carried out in triplicate. The IC_50_ was calculated graphically, using a regression analysis by plotting the extract concentration *versus* the corresponding inhibition percentage (*I*%). The *I*% for formation of AGE was calculated as follows: *I*%= (*F*_negative control_ − *F*_experimental corrected_/*F*_negative control_) × 100, where *F*_negative control_ is the fluorescence for the negative control and *F*_experimental corrected_ is the experimental fluorescence corrected for the experimental treatments.

### *In vitro* cytotoxic assay

#### Macrophage

The cytotoxic effects of EEPA and EEPB on macrophage line J774 were investigated using the MTT assay method (Mosmann 1983). Macrophages were grown according to Thomas et al. (1997) and distributed in a 96-well plate (1.5 × 10^5^ cells/well and incubated overnight [37 °C]). Adherent cells were treated with samples of the EEPA or EEPB, dissolved in PBS + DMSO (0.037%) at concentrations from 1, 10, 25, 50 or 100 μg/mL in culture medium RPMI-1640, supplemented with 10% foetal bovine serum and were then further cultured for 24 h (37 °C). Thereafter, the medium was replaced with fresh RPMI medium containing 5 mg mL^−1^ of MTT. After an additional 4 h incubation at 37 °C, the supernatant was discarded and the DMSO solution (150 μL/well) was added to each culture plate. After 15 min incubation at room temperature, the absorbance of solubilized MTT formazan product was spectrophotometrically measured, at 540 nm. Four individual wells were assayed per treatment and MTT reduction activity was determined as a percentage of control cells ([absorbance of treated cells/absorbance of untreated cells] × 100).

#### Cancer cell lines

The EEPA and EEPB (0.04–25 μg/mL) were tested for cytotoxic activity against selected human cancer cell lines: SF295 (glioblastoma), HCT-116 (colon), OVCAR-8 (ovarian), MDA-MB435 (melanoma), MX-1 (breast), MCF7 (breast), HL60 (promyelocytic leukemia), JURKAT (acute T cell leukemia), MOLT-4 (acute T cell leukemia), K562 (chronic myelogenous leukemia), PC3 (prostate) and DU145 (prostate). The extracts were incubated with the cells for 72 h. All cell lines were kindly donated by the National Cancer Institute (Bethesda, MD). Cytotoxicity was quantified through the ability of living cells to reduce the yellow dye 3-(4,5-dimethyl-2-thiazolyl)-2,5-diphenyl-2H-tetrazolium bromide (MTT, Sigma Aldrich, St. Louis, MO) to a purple formazan product and absorbance was measured at 595 nm (DTX-880, Beckman Coulter**), as described by Mosmann (1983). Also, to investigate the selectivity of samples toward a normal proliferating cell, non-cancer Chinese hamster lung fibroblasts (V79 cells), kindly provided by Dr. Henriques JAP (Federal University of Rio Grande do Sul, Porto Alegre, Brazil), and L929 mouse fibroblasts, purchased from the Rio de Janeiro Cell Bank (Federal University of Rio de Janeiro, Rio de Janeiro, Brazil) were used. Human cancer cells were maintained in RPMI 1640 medium, and the V79 and L929 murine cell lines were cultivated under standard conditions in MEM with Earle’s salts. For lymphocyte isolation, heparinized blood (from healthy, nonsmoker donors who had not taken any medication at least 15 d prior to sampling) was collected, and peripheral blood lymphocytes (PBLs) were isolated by a standard method of density-gradient centrifugation on Histopaque-1077, and had their growth stimulated with 2% phytohemaglutinin. All culture media were supplemented with 10% (cancer cells and murine fibroblasts) or 20% (lymphocytes) foetal bovine serum, 2 mmol/L glutamine, 100 μg/mL penicillin and 100 μg/mL streptomycin at 37 °C, under 5% CO_2_.

### Identification of phenolic compounds by UHPLC-MS/MS

Chromatographic analyses of the EEPs, Hex-fr, Chlo-fr and HMet-fr (1 mg/mL) were performed in an Acquity UPLC System from Waters (Milford, CT), equipped with a binary solvent delivery system, degasser, autosampler and column heater. Chromatographic separations were performed using an Acquity BEH C18 column (100 mm × 2.1 mm), with a 1.7-μm particle size from Waters. MS/MS detection was performed using a Xevo TQD tandem quadrupole mass spectrometer from Waters (Manchester, UK), coupled with an electrospray ionization interface (ESI), operating in the negative ion mode. The source parameters were capillary voltage: 4.5 kV, source temperature: 120 °C and desolvation gas temperature: 400 °C, with nitrogen flow rates of 30 and 600 L/h for the cone and desolvation gases, respectively. Mobile phase components were eluent A: ultrapure water containing 0.1% formic acid and eluent B: acetonitrile. The flow rate was 0.2 mL/min and 2 μL of samples were injected, with a linear gradient starting at 3% B, increasing to 100% B in 10 min. For identification, the multiple reaction monitoring (MRM) mode was employed to confirm the presence of phenolic compounds in the sample, along with *m*/*z* transitions of the precursor ions and product ions.

Phenolic standards (19) were monitored: apigenin, caffeic acid, cinnamic acid, coumaric acid, chlorogenic acid, ferulic acid, gallic acid, sinapic acid, syringic acid, vanillic acid, kaempferol, catechin, epicatechin, epigallocatechin, formononetin, luteolin, quercetin, rutin and 3,4-di-hydroxybenzoic acid. These compounds were chosen due to their pharmacological potential and/or have already been identified in other types of propolis.

### Characterization of propolis samples by headspace GC-MS

The headspace extraction was carried out at 40 °C for 2 h using 1.0 g of triturated propolis sample (A or B) in an amber glass tube, with a volume of 15.0 mL. The volatiles were collected with a solid phase microextraction (SPME) device of fused silica (FS) having 100 μm diameter with a 10 μm coating of a thin film (75 μm) of polydimethylsiloxane (PDMS). The analysis of the constituents was performed with a Shimadzu GC-17A gas chromatograph (Shimadzu, Tokyo, Japan) coupled to a quadrupole mass spectrometer, GCMSQP5050A. Injection in splitless mode used helium as the carrier gas, at a flow rate of 1.0 mL/min. The injector temperature was 220 °C. A fused-silica capillary column (5% phenyl–95% polydimethylsiloxane, 30 m × 0.25 mm, 0.25 μm) was employed in the separation of the compounds. The oven temperature was programed to increase from 60 °C to 240 °C, at a rate of 3 °C/min. Electron ionization (70 eV) was used and the mass scan ranged from 30 to 300 Da. The temperatures of the ion source and the GC-MS interface were 200 °C and 230 °C, respectively.

Volatile compounds were identified by analysis of the spectra, comparing these mass spectra with reported ones, mainly by analysing the features of the molecular ion and base peaks, relating them to other spectra of the database available in Wiley MS (Wiley Class 5000, sixth edition) GCM Solutions and through the comparison of Kovats indices calculated by injecting a series of standard alkanes (C7–C30), using retention indices reported in the literature. Quantitative analysis method was performed by peak area normalization method for their relative contents. The analyses were carried out in triplicate.

### Statistical analysis

All the analyses were carried out in triplicate and the results were reported as mean ± standard deviation. Analysis of variance and least significant difference tests were conducted to identify differences among the means, *p*-value <0.05 was regarded as significant. Statistical analysis was performed using GraphPad Prism 5.01 (GraphPad Software, San Diego, IL).

## Results and discussion

### EEP and fractions

The percent yields of each EEP were EEPA 26% (w/w) and EEPB 21% (w/w), with the calculation based on the weight of crude propolis. When the EEPs were subjected to liquid–liquid fractionation with hexane, chloroform and methanol, three fractions were obtained: Hex-fr, Chlo-fr and HMet-fr with yields calculated from the crude weight of EEP (Table 1). The labels A and B are related to the origin of the propolis, as previously stated.

**Table 1.** Yields of fractions after liquid/liquid extraction, total phenols content (TPC) and values of FRAP and DPPH^•^ (RSA% and IC_50_) for the EEPs and fractions.

			DPPH^•^		
Extracts	Yield (%)	TPC (mg GAE* g^−1^)	RSA (%)	IC_50_ (μg mL^−1^)	FRAP (mg TEAC** g^−1^)
EEPA	26	74.9 ± 0.7^b^	44.7 ± 3.4^b^	33.1	157.6 ± 0.2^b^
Hex-fr A	34	37.1 ± 3.5^e^	10.0 ± 0.5^e^	–	35.1 ± 3.2^e^
Chlo-fr A	54	102.4 ± 2.0^a^	20.7 ± 1.6^d^	66.0	138.7 ± 1.0^c^
HMet-fr A	11	64.3 ± 0.4^c^	52.5 ± 3.2ª	25.3	219.7 ± 3.0ª
EEPB	21	62.6 ± 1.1^c^	14.8 ± 0.8^de^	78.5	42.0 ± 3.9^d^
Hex-fr B	8	44.0 ± 0.5^d^	7.7 ± 0.9^e^	–	20.1 ± 0.4^f^
Chlo-fr B	46	45.3 ± 0.4^d^	28.3 ± 0.2^c^	55.8	42.0 ± 0.7^d^
HMet-fr B	37	77.5 ± 4.7^b^	38.1 ± 1.1^b^	34.2	153.8 ± 2.4^b^

EEPA was obtained from propolis of Ribeira do Pombal and EEPB from propolis collected at Tucano. Hex-fr: hexane fraction; Chlo-fr: chloroform fraction; HMet-fr: hydromethanol fraction.

*Gallic acid equivalents. **Trolox equivalent antioxidant capacity.

^1^Values are mean ± SD. Means with different letters within a column are significantly different (*p* < 0.05).

### Determination of TPC and antioxidant capacity

Table 1 displays the values obtained for the TPC, using the FC method and the antioxidant capacity obtained through DPPH^•^ and FRAP methods.

The FC assay is often used as a measure of TPC in natural products. Generally, there is a positive correlation between this method and other antioxidant capacity measurements. However, FC is known to be non-selective to phenolic compounds, as it can be reduced by non-phenolic compounds such as vitamin C and reducing agents (Prior et al. 2005; de Oliveira et al. 2009). The TPC values ranged from 37.1 to 102.4 mg GAE/g for sample A and between 44.0 and 77.5 mg GAE/g for sample B.

EEPA, Chlo-fr A and HMet-fr B showed the highest TPC: 74.9, 102.4, and 77.5 mg GAE/g, respectively. Hexane extracts showed the lowest values of TPC, as expected, since hexane is less efficient for extraction of polar phenolic compounds from propolis.

DPPH^•^ and FRAP methods are used for the evaluation of antioxidant capacity (Prior et al. 2005; de Oliveira et al. 2009). DPPH^•^ is a stable radical in solution and this assay is routinely, used for assessment of free radical scavenging potential of antioxidants, through single electron transfer (SET) or by radical quenching via H atom transfer (HAT). The FRAP assay measures the ability of antioxidants to reduce ferric ion in the complex [Fe(III)(TPTZ)_2_]^3+^ to [Fe(II)(TPTZ)_2_]^2+^ through electron transfer and cannot detect compounds that act by HAT (Duraisamy et al. 2003; Prior et al. 2005; Craft et al. 2012; Castro et al. 2014).

HMet-frA and EEPA, through analysis by the DPPH^•^ method, showed higher radical sequestrating ability with values of 52.5 and 44.7%, respectively, together with HMet-fr B, with a value of 38.1%, statistically similar to EEPA (Table 1). The other fractions of samples A and B showed lower values. The IC_50_ is related to the concentration that can reduce DPPH^•^ by 50%. Thus, the lower the concentration, the more efficient the sample is. HMet-fr A showed the lowest IC_50_ value, of 25.3 μg/mL, followed by EEPA and HMet-fr B with 33.1 and 34.2 μg/mL, respectively (Table 1).

With regard to the FRAP method, HMet-fr A, EEPA and HMet-fr B also showed higher values, 219.7, 157.6 and 153.8 mg TE/g dry extract, respectively.

From Table 1, it is possible to observe that EEP and the more polar fractions exhibit higher antioxidant capacity and that extracts from sample A are more promising as antioxidants than those from sample B.

Table 2 reports antioxidant capacity data of different samples of propolis. Some literature results could not be included, since the results were expressed differently. For instance, when caffeic acid was used instead of gallic acid as a standard compound. It is possible to observe (Table 2) that the values of TPC, FRAP and DPPH^•^ (IC_50_) are close to most of the values found in the literature for other propolis samples, with the exception of some samples from Turkey and Brazil (Group 6) that presented lower results.

**Table 2.** Comparative results of TPC, DPPH^•^ and FRAP among extracts of propolis from different regions.

Propolis samples (local)	TPC (mg GAE g^−1^)	DPPH^•^ IC_50_ (μg mL^−1^)	FRAP (mg TE g^−1^)	References
Bahia, Brazil	37.1–102.4	25.3–78.5	20.1–219.0	Present work
Sergipe, Brazil, red	151.5	270.1	–	Frozza et al. (2013)
Brazil, green	–	–	674.0	Skaba et al. (2013)
Canada	65.9–199.3	26.4–101.7	–	Cottica et al. (2015)
France	238.6–292.1	–	–	Boisard et al. (2014)
Brazil, group 12	169.6	–	–	Cabral et al. (2012)
Brazil, group 6	14.8	–	–	Cabral et al. (2012)
Minas Gerais, Brazil, green	–	24.1	–	Szliszka et al. (2013)
China	174.7	32	–	Yang et al. (2011)
Turkey	9.2–48.7	–	24.1–59.5	Barlak et al. (2011)

### Lipid peroxidation measurements

The scavenging free radical capacities do not necessarily correlate with the ability of inhibiting lipid peroxidation. Thus, it is important to evaluate this protective effect through a specific test (Ferreira et al. 2013).

The assay was performed using a peroxyl radical-mediated lipid peroxidation membrane model (soy lecithin unilamellar liposomes), loaded with the peroxyl radical-sensitive fluorescent probe C11-BODIPY_581/591_, as described in Materials and methods section.

**Figure 2.**
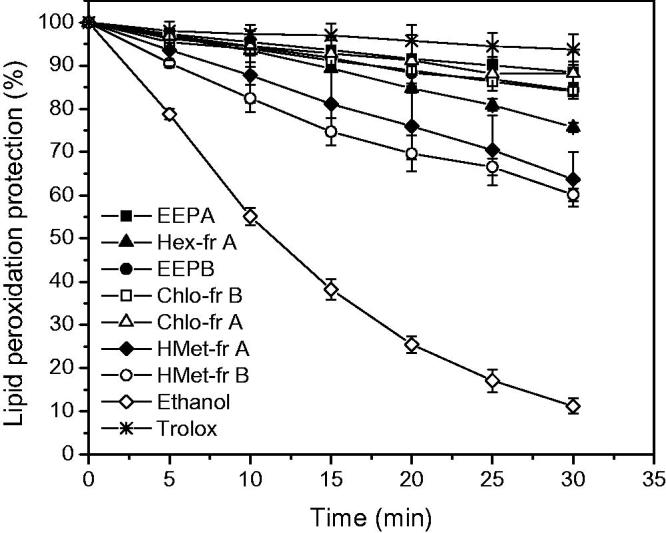
Lipid peroxidation protection (%) provided by the ethanol extracts of propolis and fractions (25 μg mL^−1^), positive control (Trolox 100 μM) and the negative control, ethanol. Liposome plus C11-BODIPY_581/591_ were added in all cases.

Figure 2 displays lipid peroxidation protection (%) as a function of time (total time of 30 min) for the whole samples and fractions of propolis, in comparison with the positive and negative controls.

It can be observed that in the blank, in the absence of an antioxidant, liposomal lipid peroxidation induced by AAPH (generator of peroxyl radical) occurs, which causes the fluorescence decay. The positive control, Trolox^^®^^, as might be expected, inhibited lipid peroxidation, offering about 95% membrane protection.

All studied extracts showed protection of the membrane from lipid peroxidation. A lower protection of approximately 60% was observed in the Hex-fr B, HMet fr-B and HMet-fr A extracts, while the EEPA, Chlo-fr A, EEPB and Chlo-fr B extracts offered membrane protection similar to Trolox^^®^^ within 15 min and, after 30 min, showed about 90% protection, and thus proved to be the most promising result in this study. Comparing the values of Table 1 with those results of lipid peroxidation, it is possible to conclude that EEPA and EEPB show better results than their fractions.

### Anti-glycation activity

There is strong evidence of the involvement of AGEs in the pathophysiology of degenerative chronic diseases. Their incidence and growing concern, around the world, have stimulated research toward the discovery of natural and synthetic compounds capable of inhibiting their potentially harmful effects on health (Ramkissoon et al. 2012a; Barbosa et al. 2016).

The anti-glycation activity is related to the ability to prevent AGE formation (Chinchansure et al. 2015), determined by measuring the fluorescence intensity of BSA-glucose or fructose solutions in the presence or absence of the extracts (Ramkissoon et al. 2013).

Compounds with antioxidant activity, such as quercetin, gallic acid, luteolin, among others, have been proven to exhibit anti-glycation effects (Chinchansure et al. 2015). As such, *in vitro* anti-glycation activities for EEPA and EEPB were evaluated.

Anti-glycation activity of EEPA and EEPB represented by IC_50_ values was 16.5 ± 0.4 and 19.2 ± 1.1 μg/mL, respectively. For the pure compound, aminoguanidine, used as an anti-glycation standard, the IC_50_ value was 7.7 ± 0.6 μg/mL and this value was only 2.5 times lower than the values for the crude EEPA and EEPB extracts. Our results are better than the ones obtained by Boisard et al. (2014), who studied anti-glycation activity of French poplar propolis and found IC_50_ = 30 μg/mL.

Some studies have already evaluated the action of propolis on the inhibition of glycation and as a complementary treatment of diabetes mellitus. Sahebi and Divsalar (2016) investigated the effects of the ethanolic extract of Iranian propolis (EEIP), on the glycation of human haemoglobin by glucose. They demonstrated that haemoglobin glycated by glucose reduced the free amino group content and increased amyloid structures and haeme degradation. The utilization of EEIP prevented these changes and decreased the extent of glycation in a concentration-dependent manner.

El-Sayed et al. (2009) have shown that Brazilian green propolis extract offered promising antidiabetic and hypolipidemic effects in streptozotocin-induced diabetic rats. Oladayo (2016) studied the effect of ethanolic extract of Nigerian propolis on plasma glucose, showing that it decreased glycated haemoglobin A1c (HbA1c) and some blood lipids such as very low-density lipoprotein (VLDL) and high-density lipoprotein (HDL), in type 1 diabetic rats. They observed a significant reduction in glycaemia increase, in the rate of HbA1c formation, and in ameliorated diabetic dyslipidaemia, shown by increasing HDL levels.

The excellent anti-glycation activity of the present EEPA and EEPB makes them exciting candidates for reducing protein glycation, acting as a supplementary therapy for diseases associated with excessive accumulation of AGE, especially for complications of diabetes mellitus (Babu et al. 2013; Singh et al. 2013). Additional studies are, however, mandatory to identify the mechanism of biological action, as well as the *in vivo* response in suitable model organisms, before a clinical application.

### Cell viability assay

The results revealed that both anti-glycation and antioxidant activities of EEPA and EEPB (Table 1) indicate them to be promising natural products for use as nutraceuticals. A preliminary cell viability assay was performed to determine the cytotoxic effect of different concentrations of the extracts, looking for safety of the present propolis.

**Figure 3.**
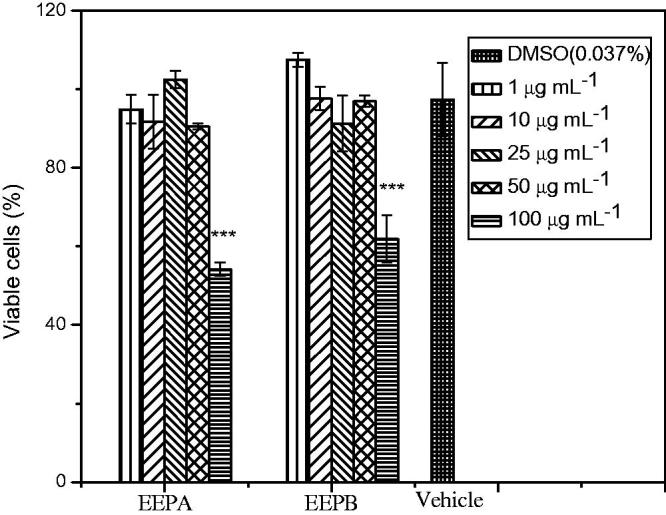
Effect of EEPA and EEPB on viability of macrophages (lineage J774), using MTT assay. Cells were treated with EEPA and EEPB (1, 10, 25, 50 or 100 μg mL^−1^) and exposed for 24 h. Data were expressed as % viable cells and were shown as mean ± SEM. ***p < 0.001 versus other concentrations. Negative control was the solvent used to dissolve the extracts.

Cytotoxicity of EEPA and EEPB extracts on murine macrophages, important cells of the immune system, was evaluated, by observing the reduction of MTT. It is possible to observe, in Figure 3, that the treatment of these cells for 24 h with the extracts up to a concentration of 50 μg/mL did not decrease their viabilities. However, when cells were treated with the extracts at a concentration of 100 μg/mL, there was a significant reduction (*p* < 0.001) in the viability of macrophages by 55% and 62%, respectively.

Szliszka et al. (2013) investigated the ethanolic extract of Brazilian green propolis and obtained a similar result. The green propolis extract did not influence cell viability and did not exert a cytotoxic effect at concentrations <50 μg/mL, so this is the recommended concentration for further studies.

Cytotoxicity toward human cancer cell lines was also investigated. Table 3 lists the results for EEPA and EEPB. The evaluation was conducted in accordance with the protocol of the National Cancer Institute (NCI), which recommends that IC_50_ values ≤30 μg/mL should be considered significant for crude extracts of plant origin as well as IC_50_ values ≤4 μg/mL for pure substances (Geran et al. 1972).

**Table 3.** Cytotoxic activity expressed by IC_50_ in μg/mL (95% CI) of EEPA and EEPB, in cancer cell lines, after 72 h exposure, obtained by nonlinear regression for all cell lines from three independent experiments.

Cell lines	EEPA	EEPB
HL-60	15.42 (13.69–17.84)	19.24 (17.51–21.03)
JURKAT	17.30 (15.76–19.22)	14.85 (13.59–16.21)
MOLT-4	14.06 (12.83–15.74)	16.28 (15.73–17.52)
K562	22.18 (21.94–23.47)	20.37 (19.82–21.56)
PC3	>25	>25
DU145	>25	>25
HCT-116	>25	>25
MCF7	>25	>25
OVCAR-8	>25	>25
MDA-MB435	>25	>25
SF295	>25	>25
MX-1	>25	>25

The evaluated samples showed cytotoxicity only on leukemia cell proliferation, showing a certain selectivity. Interestingly, the two samples studied had no toxic effects on non-tumor cells (PBMC, V79 and L929), with IC_50_ > 25 μg/mL.

### Phenolic compounds identified in extracts of propolis from samples A and B

The identification of phenolic compounds in propolis extracts (1 mg/mL) was performed using UHPLC-ESI-MS/MS. Qualitative results are presented in Table 4. The identification of compounds was achieved by matching retention times (*t*_R_) and MS/MS fragmentation patterns, with authentic reference standards. A total of 19 standard compounds in different samples of propolis were monitored, from which, 12 were identified in the present extracts.

**Table 4.** Compounds identified by UHPLC-ESI-MS/MS in extracts of propolis samples.

Compound	RT (min)	Molar mass (g/mol)	[M–H]^−^ (*m*/*z*)	Confirmation transition (*m*/*z*)	Extracts
Gallic acid	0.77	170.1	169.0	78.8/125.0	EEPA, HMet-fr A, Chlo-fr A, Hex-fr A, EEPB, HMet-fr B.
Chlorogenic acid	1.45	354.3	353.0	84.6/191.1	EEPA, HMet-fr A, EEPB, HMet-fr B.
Caffeic acid	2.61	180.2	179.0	116.9/135.0	EEPA, HMet-fr A, Chlo-fr A, EEPB, HMet-fr B, Chlo-fr B.
Coumaric acid	3.55	164.2	162.8	92.8/118.9	EEPA, HMet-fr A, Chlo-fr A, Hex-fr A, EEPB, HMet-fr B, Chlo-fr B, Hex-fr B.
Ferulic acid	4.22	194.2	192.9	78.8/125.0	EEPA, HMet-fr A, Chlo-fr A, EEPB, HMet-fr B, Chlo-fr B.
Rutin	4.65	610.5	609.2	300.2/271.2	EEPA, HMet-fr A, Chlo-fr A, EEPB, HMet-fr B.
Quercetin	4.66 6.10	302.2	301.0	151.0/179.1	EEPA, HMet-fr A, Chlo-fr A, Hex-fr A, EEPB, HMet-fr B, Chlo-fr B, Hex-fr B.
Luteolin	6.07	286.2	285.0	103.0/133.0	EEPA, HMet-fr A, Chlo-fr A, EEPB, HMet-fr B, Chlo-fr B
Kaempferol	6.08	286.2	285.0	92.8/187.2	EEPA, HMet-fr A, EEPB, HMet-fr B, Chlo-fr B, Hex-fr B.
Cinnamic acid	6.21	148.2	146.9	88.9/103.0	EEPA, HMet-fr A, Chlo-fr A, EEPB, Chlo-fr B.
Apigenin	6.54	270.2	269.0	117.0/151.0	EEPA, HMet-fr A, EEPB, HMet-fr B, Chlo-fr B, Hex-fr B.
Formononetin	7.26	268.2	267.2	223.3/252.2	EEPA, Chlo-fr A, Hex-fr A, EEPB, HMet-fr B, Chlo-fr B, Hex-fr B.

Several earlier studies have confirmed the variety in the chemical composition of different propolis samples. Righi et al. (2013) characterized the chemical profile of the propolis from different Brazilian locations. They have identified caffeic acid, quinic acid, naringenin, quercetin, ferulic acid, luteolin, apigenin and chrysin in propolis from Paraná, Minas Gerais, Goias, Bahia, and Piauí. Fernandes-Silva et al. (2013) analyzed the constituents of the Brazilian green propolis from Minas Gerais and Paraná states. All samples showed prenylated phenylpropanoids, such as artepillin C, caffeoylquinic acid and flavonoids, such as kaempferide. However, luteolin 5-*O*-methyl ether was detected only in samples from Paraná. From brown propolis, classified as Propolis Type 6, collected in Entre Rios, Bahia, methyl cinnamate, sitosterol cinnamate and ananixanthone were identified (Dos Santos et al. 2017).

In Chinese propolis, Yang et al. (2013) identified rutin, quercetin, genistein, curcumin, luteolin and galangin. Morlock et al. (2014) reported the presence of coumaric acid, chrysin, pinocembrin, galangin, apigenin and narigenin in German propolis. Piccinelli et al. (2013) found kaempferol, apigenin and derivatives of caffeic acid in Algerian propolis. Quercetin was found in Indian propolis (Thirugnanasampandan et al. 2012).

Caffeic, coumaric and ferulic acids have been shown to inhibit the proliferation of tumor cells of human lung (A549) and adenocarcinoma (HT29-D4), and significantly reduced superoxide radical anion production by these cells (Bouzaiene et al. 2015). Gallic acid presented inhibitory effect on proliferation and induction of apoptosis in human breast carcinoma (MCF-7) cells (Wang et al. 2014).

Compounds such as caffeic and chlorogenic acids are inhibitors of AGEs formation in BSA/glucose (fructose) (Chinchansure et al. 2015) and BSA/methylglioxal systems (Gugliucci et al. 2009). Gallic acid and quercetin had shown inhibitory effects on the production of Amadori products (Chinchansure et al. 2015).

It is possible to observe that there are some similarities between compounds identified in this study (Table 4) and compounds identified in propolis from different regions. Although belonging to different geographical origins, some compounds are of broad occurrence among samples of propolis, while others are specific and are called chemosystematic markers.

### Volatile profile by GC-MS

GC analyses were performed with the two crude samples of propolis. Twenty-four volatile compounds were identified by GC-MS, with eight of them, present in both samples. Table 5 lists the volatile constituents identified according to their chemical classes.

**Table 5.** Volatile components identified in propolis crude samples (A and B).

			Area (%)	
Compound	Molar mass	K.I.	Sample A	Sample B
*Alcohols*				
2-Phenylethanol	122.16	1113	0.6	–
*Aldehydes*				
Octanal	128.21	1003	1.8	–
Nonanal	142.24	1104	6.3	0.8
*Aliphatic and aromatic acids*				
Acetic acid	60.05		13.2	–
Benzoic acid	122.12	1172	1.2	–
Hexanoic acid	116.15	999	5.8	0.8
*Ketones*				
6-Methyl-5-hepten-2-one	126.20	987	1.1	0.5
2-Nonanone	142.23	1092	1.2	–
*Terpenoids*				
α-Bergamotene	204.35	1447	–	6.0
α-Bisabolol	222.36	1696	–	37.0
α-Copaene	204.35	1390	0.8	0.3
α-Cubebene	204.35	1361	1.0	0.5
α-Curcumene	202.33	1491	–	5.0
α-Farnesene	204.35	1513	2.8	–
α-Gurjenene	204.35	1417	0.7	–
α-Pinene	136.23	940	5.6	–
β-Bourbonene	204.35	1390	0.4	–
β-Caryophyllene	204.35	1434	2.3	1.9
β-Pinene	136.23	982	2.1	–
δ-Cadinene	204.35	1537	0.5	2.3
Limonene	136.23	1033	7.1	6.9

The results showed a quite diversified volatile chemical composition, with acetic acid, α-pinene, limonene, nonanal, 1,8-cineole and spathulenol, standing out as major constituents for sample A, and α-bisabolol, limonene, α-bergamotene and ar-curcumene for sample B. The identified compounds have already been found in other propolis samples.

In other samples of Brazilian propolis, with different origins, α-pinene, β-pinene (Torres et al. 2008; Simionatto et al. 2012; Pellati et al. 2013; Kaškonienė et al. 2014) and 1,8-cineole (Torres et al. 2008; Simionatto et al. 2012) were found as the major volatile compounds.

Bankova et al. (2014) reported the main volatile constituents of propolis from different geographic regions. They had shown that the main constituents of European propolis are sesquiterpenes, followed by aromatic compounds. In Chinese propolis, from 23 regions of China, acetic acid, 2-phenylethyl acetate and naphthalene were found. Oxygenated hydrocarbons, oxygenated sesquiterpenes, monoterpenes (α-terpinene and α-terpineol), aromatic alcohols and esters were the major volatile compounds of Turkish propolis. In samples of Brazilian green propolis, the main volatile constituents are nerolidol, β-caryophyllene, spatulenol, δ-cadinene and monoterpenes such as α-pinene and β-pinene, some of them also found in the present investigation.

Several studies attribute antioxidant and/or anti-glycation properties to phenolic compounds, flavonoids and terpenes (Sri Harsha et al. 2013; Chinchansure et al. 2015; Sadowska-Bartosz and Bartosz 2015). Additionally, it is possible to correlate antioxidant and anti-glycation properties.

Naringenin, kaempferol, quercetin and rutin also exhibited protective effects against glycation. Luteolin and apigenin exhibit inhibition *in vitro* of AGEs by trapping reactive methylglyoxal. Limonene exhibited antidiabetic, anti-glycation and antioxidant properties. All of them occur in the presently investigated propolis samples. Terpenoids such as labadiiene exhibited potent inhibitory activities on the formation of fructosamine adducts and α-dicarbonyl compounds similar to that of the flavonoids quercetin and rutin. Triterpenic oleanolic acid showed antiglycation activities greater than aminoguanidine. Another triterpenic acid, nolic acid, is effective in preventing the formation of HbA1c, ROS, AGEs and oxidative stress signalling. β-Carotene, a tetraterpenoid, showed inhibitory effects on the formation of AGEs and prevented secondary structural changes in BSA, resulting from glycation (Chinchansure et al. 2015).

These results suggest the promising functional nature of these natural products. Anti-glycation assays, reported in the current study, may provide a new mechanism by which polyphenol-rich natural products can have a positive effect on human health (Lo et al. 2006; Chen et al. 2012).

## Conclusions

This work has reported, for the first time, a detailed study of propolis produced in the Tucano and Ribeira do Pombal, regions of Bahia. The samples show good antioxidant and anti-glycation capacities, cytotoxicity against several cancer cell lines, such as HL-60, JURKAT and MOLT-4, and a significant number of biologically active compounds, suggesting their potential use for pharmaceutical and medicinal purposes, targeting ageing and ROS- and AGE-biochemically based diseases. Along with the present significant chemical results and the related beneficial health aspects, there is an additional social and economic value for both producers and other local people.

## Supplementary Material

Marilia_Fonseca_Goulart_et_al_supplemental_content.zip
