# Supplementary material for: Polyphenol profile by UHPLC-MS/MS, anti-glycation, antioxidant and cytotoxic activities of several samples of propolis from the northeastern semi-arid region of Brazil
Source: Pharm Biol. 2017 Jun 20;55(1):1884–93. doi: 10.1080/13880209.2017.1340962 (PMC6131762; doi:10.1080/13880209.2017.1340962)
Supplement: Marilia_Fonseca_Goulart_et_al_supplemental_content.zip [file IPHB_A_1340962_SM4609.zip › Marilia Fonseca Goulart et al supplemental content.docx]

**Supplementary Information**

Polyphenol profile by UHPLC-MS/MS, anti-glycation, antioxidant and cytotoxic activities of several samples of propolis from the northeastern semi-arid region of Brazil

Jadriane A. Xavier^a^, Iara B. Valentim^b^, Fabiana O. S. Camatari^a^, Alberto M. M. de Almeida^c^, Henrique F. Goulart^d^, Jamylle N. S. Ferro^e^, Emiliano O. Barreto^e^,

Bruno Coelho Cavalcanti^f^, Carla B. G. Bottoli^g^, Marília O. F. Goulart ^a*^

*^a^ Instituto de Química e Biotecnologia, Universidade Federal de Alagoas (UFAL), 57072-970 Maceió, AL, Brazil.*

*^b^ Instituto Federal de Educação, Ciência e Tecnologia de Alagoas (IFAL), Rua Mizael Domingues, 75, Centro, 57020-600, Maceió, AL, Brazil.*

*^c^ Empresa Baiana de Desenvolvimento Agrícola S.A (EBDA), 40170-110 Salvador, BA, Brazil.*

*^d^ Laboratório de Pesquisas em Recursos Naturais, Centro de Ciências Agrárias (CECA), UFAL, BR 104, Km 85, s/n, 57100-000, Rio Largo, AL, Brazil.*

*^e^ Laboratório de Biologia Celular, UFAL, 57072-970, Maceio, AL, Brazil.*

*^f^ Departamento de Fisiologia e Farmacologia, Laboratório Nacional de Oncologia Experimental, Universidade Federal do Ceará, Fortaleza, CE, 60416-000,* *Brazil.*

*^g^ Instituto de Química, Universidade de Campinas, P.O. Box 6154, 13083–970, Campinas, SP, Brazil.*

In this study, twelve phenolic compounds (Figure S1) were identified by the use of UHPLC-MS/MS, which combines the high separation power of ultra high performance liquid chromatography (UHPLC), with multiple reaction monitoring (MRM) mode, which performs monitoring of precursor-to-product ion transitions. The total ion chromatogram (TICs) for each identified compound in EEPA and EEPB are respectively shown in Figure S2 and Figure S3 and the fragmentation parameters were optimized for each standard (Table S1).

**Figure S1.** Structures of the compounds identified by UPLC-ESI-MS/MS in extracts of propolis samples


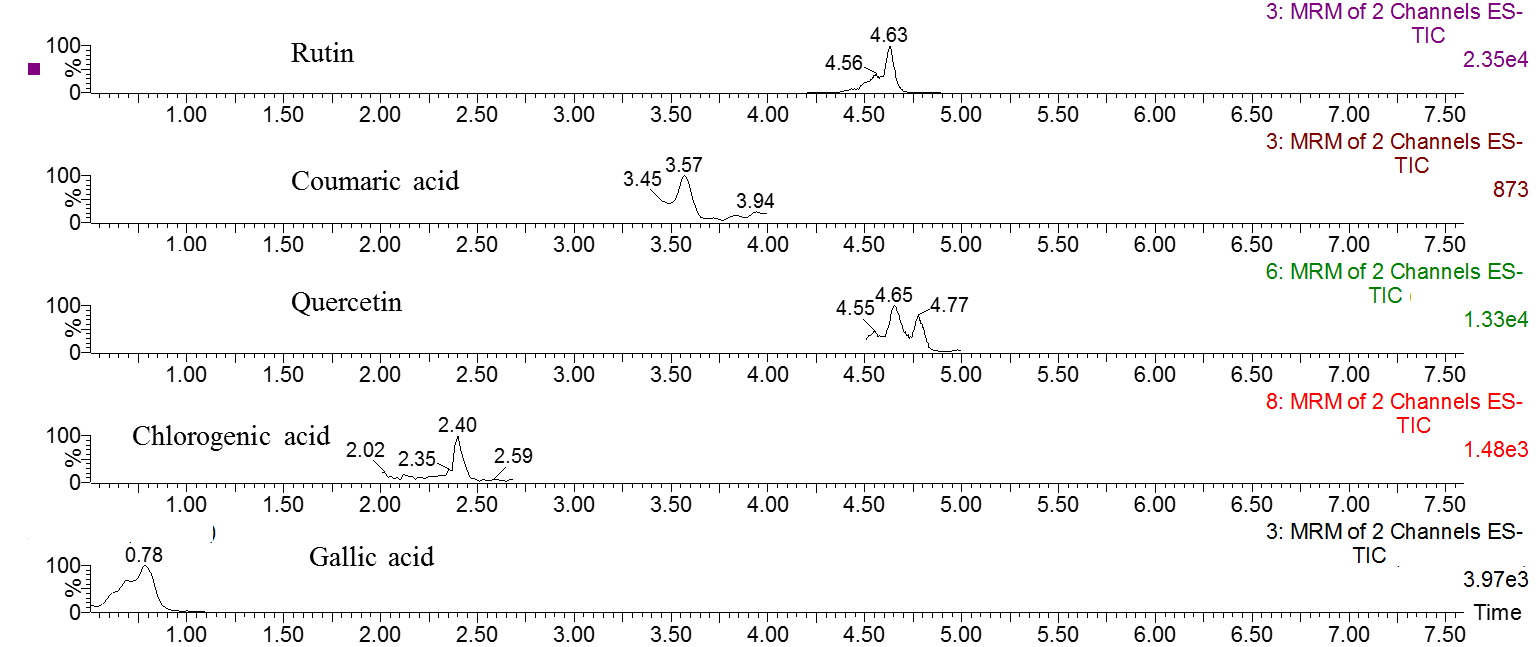

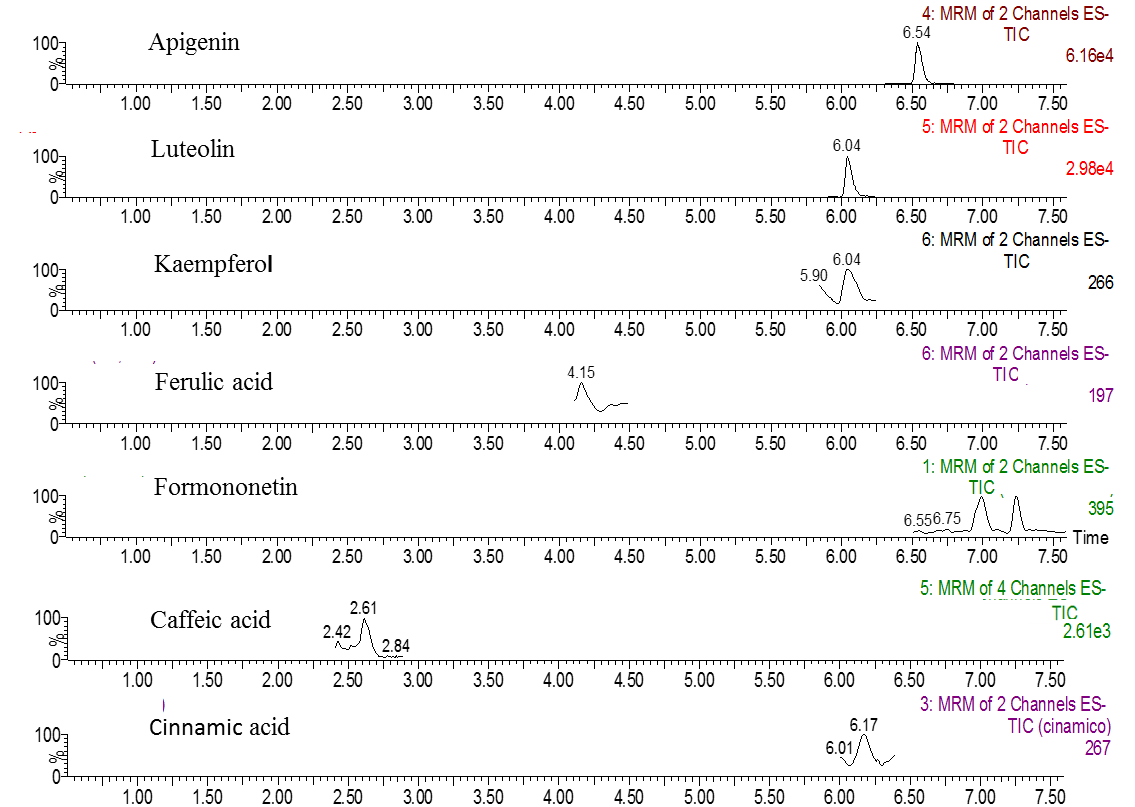


### Figure S2. [Total Ion Chromatogram (TIC)](http://www.shimadzu.com/an/total_ion.html) for EEPA.


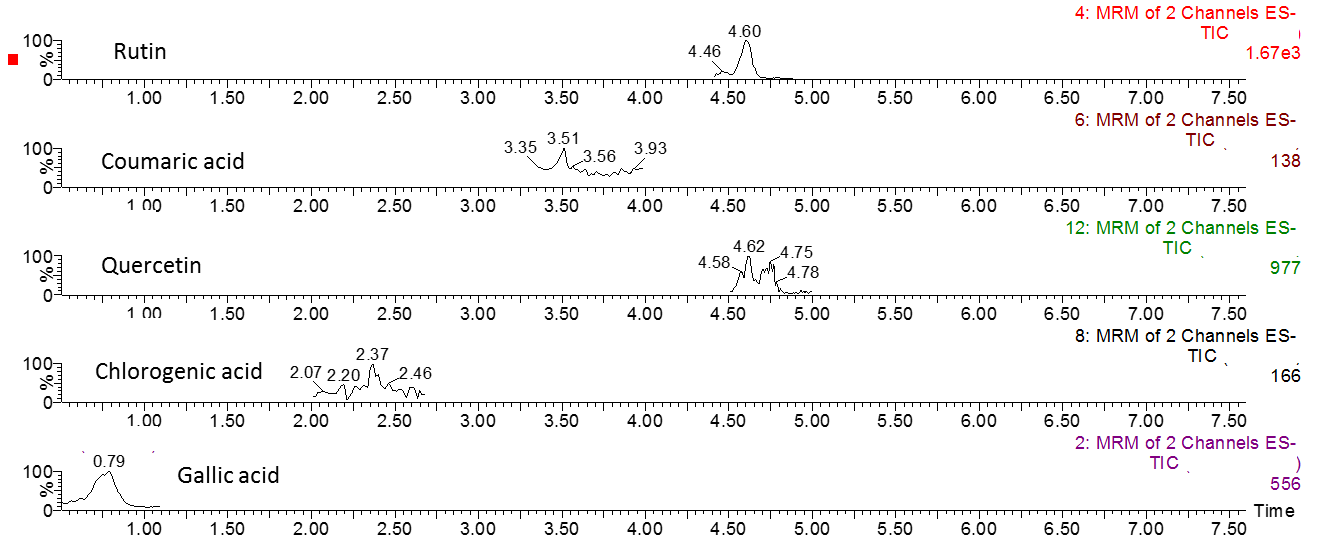

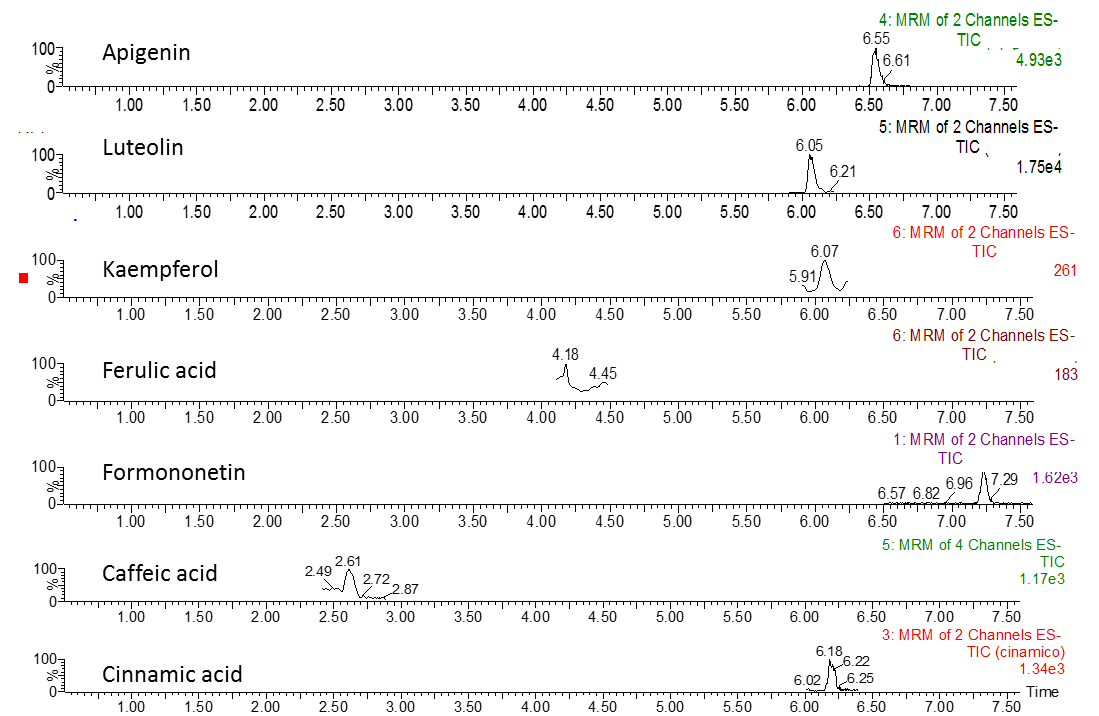


### Figure S3. [Total Ion Chromatogram (TIC)](http://www.shimadzu.com/an/total_ion.html) for EEPB.

**Table S1.** MRM transitions, ionization and fragmentation parameters used in UPLC-ESI-MS/MS.

| Compound | MRM  quantification | | Cone voltage (V) | | Collision energy (eV) | | Confirmation transition (m/z) | | Cone voltage (V) | | Collision energy (eV) | |  |
| --- | --- | --- | --- | --- | --- | --- | --- | --- | --- | --- | --- | --- | --- |
| Gallic acid | | 169.0 > 125.0 | | 25 | | 15 | | 169.0 > 78.8 | | 25 | | 20 | |
| Chlorogenic acid | | 353.0 > 191 | | 30 | | 15 | | 84.6 > 191.1 | | 30 | | 45 | |
| Caffeic acid | | 179.0 > 135.0 | | 30 | | 15 | | 179.0 > 116.9 | | 30 | | 35 | |
| Coumaric acid | | 162.8 > 118.9 | | 35 | | 15 | | 162.8 > 92.8 | | 35 | | 35 | |
| Ferulic acid | | 192.9 > 125.0 | | 30 | | 10 | | 192.9 > 78.8 | | 30 | | 15 | |
| Rutin | | 609.2 > 271,2 | | 60 | | 40 | | 609.2 > 300.2 | | 60 | | 50 | |
| Quercetin | | 301.0 > 179.1 | | 45 | | 20 | | 301.0 > 151.0 | | 45 | | 20 | |
| Luteolin | | 285.0 > 151.0 | | 50 | | 25 | | 285.0 > 133.0 | | 50 | | 30 | |
| Kaempferol | | 285.0 > 187.2 | | 48 | | 30 | | 285.0 > 92.8 | | 48 | | 34 | |
| Cinnamic acid | | 146.9 > 103.0 | | 25 | | 10 | | 146.9 > 88.9 | | 25 | | 20 | |
| Apigenin | | 269.0 > 151.0 | | 50 | | 25 | | 269.0 > 117.0 | | 50 | | 35 | |
| Formononetin | | 267.2 > 252.2 | | 35 | | 20 | | 267.2 > 223.3 | | 35 | | 30 | |
